# Mental health circumstances among health care workers and general public under the pandemic situation of COVID-19 (HOME-COVID-19)

**DOI:** 10.1097/MD.0000000000020751

**Published:** 2020-06-26

**Authors:** Surapon Nochaiwong, Chidchanok Ruengorn, Ratanaporn Awiphan, Yongyuth Ruanta, Waraporn Boonchieng, Sirisak Nanta, Woravut Kowatcharakul, Wanida Pumpaisalchai, Penkarn Kanjanarat, Pajaree Mongkhon, Kednapa Thavorn, Brian Hutton, Nahathai Wongpakaran, Tinakon Wongpakaran

**Affiliations:** a Department of Pharmaceutical Care; b Pharmacoepidemiology and Statistics Research Center (PESRC), Faculty of Pharmacy; c Faculty of Public Health, Chiang Mai University, Chiang Mai; d Maesai Hospital, Maesai District, Chiang Rai Province; e Sansai Hospital, Sansai District; f Suanprung Psychiatric Hospital, Chiang Mai; g Division of Pharmacy Practice, Department of Pharmaceutical Care, School of Pharmaceutical Sciences, University of Phayao, Phayao, Thailand; h Ottawa Hospital Research Institute, Ottawa Hospital; i Institute of Clinical and Evaluative Sciences, ICES uOttawa; j School of Epidemiology and Public Health, Faculty of Medicine, University of Ottawa, Ottawa, Ontario, Canada; k Department of Psychiatry, Faculty of Medicine, Chiang Mai University, Chiang Mai, Thailand.

**Keywords:** coronavirus, COVID-19, general population, health care workers, mental health, psychosocial problems

## Abstract

**Background:**

After the spread of the coronavirus disease 2019 (COVID-19) globally, upgraded quarantine and physical distancing strategy, strict infection measures, and government's strict lockdown have been abided to confront the spread of the COVID-19 in Thailand. During the COVID-19 pandemic, concerns about the mental health and psychosocial problems among health care workers and the general population are now arising. Yet, information on mental health and psychosocial problems among health care workers and the general population have not been comprehensively reported in Thailand. As such, we conduct a cross-sectional study, a national online survey to describe the short- and long-term consequences of the COVID-19 pandemic on mental health and psychosocial problems among health care workers and the general population in Thailand.

**Methods:**

This study is a repeated cross-sectional study, an open online voluntary national-based survey during the wave I (April 21–May 4, 2020) follow-up in the wave II (August 3–16, 2020), wave III (November 15–28, 2020), and a 1-year follow-up survey (wave IV: April 21–May 4, 2021) in Thailand. Health care workers at the hospitals and the adult general population will be invited to participate in the online survey via the SurveyMonkey that limits one-time participation per unique internet protocol address. The target sample size of at least 1182 health care workers and 1310 general populations will be required to complete the online survey for each wave of the survey. Sociodemographic characteristics and a set of measurement tools for mental and psychosocial problems for each subcohort including depression, anxiety, stress, resilient copings, neuroticism, perceived social support, wellbeing, somatic symptoms, insomnia, burnout (for healthcare workers), and public stigma toward COVID-19 infection (for the general population) will be collected. For all estimates of prevalence, we will weigh data for all wave analyses under the complex design of the survey. Subgroup analyses stratified by key characteristics will also be done to analyze the proportion differences. For the repeated cross-sectional survey, we will combine the data from the wave I to wave IV survey to analyze changes in the mental health status. We will perform multilevel logistic regression models with random intercepts to explore associations with individual-level and region-level/hospital-level predictors. We also plan to perform an ancillary systematic review and meta-analysis by incorporating data from our findings to all available evidence.

**Results:**

Our findings will provide information on the short- and long-term mental health status as well as the psychosocial responses to the COVID-19 outbreak in a national sample of health care workers and the general population in Thailand.

**Conclusion:**

This prospective, nationally based, a repeated cross-sectional study will describe the mental health status and psychosocial problems among health care workers and the general population in Thailand during the COVID-19 pandemic.

**Ethics and dissemination:**

Ethical approval for the study was obtained from the Faculty of Public Health and Faculty of Pharmacy, Chiang Mai University. The findings will be disseminated through public, scientific, and professional meetings, and publications in peer-reviewed journals.

**Thai Clinical Trials Registry (TCTR) registration number:**

TCTR20200425001.

## 1 Introduction

After the spread of the coronavirus disease 2019 (COVID-19) globally, a patient was first reported, as a result, a community transmission has been rapidly evolved in Thailand. As of April 26, 2020, the COVID-19 caused 51 deaths of the 2922 confirmed cases, and 101 health care workers have been infected in both central and regional parts of Thailand.^[1]^ Based on the information concerning the confirmed cases, upgraded quarantine and physical distancing strategy, strict infection measures, and government's strict lockdown have been abided to confront the spread of the COVID-19 in Thailand.

Facing this large-scale infectious public health event, the ever-increasing number of confirmed and suspected cases, heavy workloads, negative emotions, depletion of personal protection equipment, lack of specific drugs, and feelings of being inadequately supported may all contribute the mental burden of health care workers.^[2]^ Meanwhile, confirmed and suspected cases of COVID-19 may experience fear of disease progression and the transmission to their family and friends. Concerns about the mental health and psychosocial problems among the general population are now arising owing to fear of contagion, perception of danger, widespread media coverage, financial problems, and unemployed related to the COVID-19 outbreak.^[3]^

Taken together, the infectious disease outbreaks, including the COVID-19 pandemic, can lead to the feeling of uncertainty, anxiety, and fear, and stigmatization in both health care workers and the general population who are under quarantine or at home. As such, it was postulated that the quarantined individuals, health care workers, as well as the general population are at risk of developing psychological distress and other mental health problems. To provide more advanced mental health care at the time of the pandemic, it is necessary to understand the mental health status and psychosocial problems of health care workers and the general population.

To our knowledge, however, little is known about how the COVID-19 pandemic may affect the mental health status and psychosocial problems of the health care workers and the general population in Thailand. Therefore, we will conduct a repeated cross-sectional study to understand the short- and long-term responses to the COVID-19 outbreak, examine the prevalence of mental health status, psychosocial related to the COVID-19 pandemic, and identify associated factors among a national sample of health care workers and the general population in Thailand.

## 2 Objectives of the survey

1. To examine the prevalence of mental health and psychosocial problems among health care workers and the general population in Thailand during the COVID-19 pandemic.
2. To document the changes over a 1-year follow-up of psychological response to a national infectious public health event.
3. To identify early and late predictors of mental health status in response to the COVID-19 outbreak.
4. To establish the prevalence of mental health status among health care workers and the general population worldwide during the COVID-19 pandemic.

## 3 Methods

### 3.1 Study design and time-period

The Health Outcomes and Mental Health Care Evaluation Survey: Under the Pandemic Situation of COVID-19 (HOME-COVID-19) is a repeated cross-sectional study, an open online voluntary national-based survey conducted during the wave I (April 21, 2020 to May 4, 2020) follow-up in the wave II (August 3, 2020 to August 16, 2020), wave III (November 15, 2020 to November 28, 2020), and wave IV—a 1-year follow-up survey (closed out: April 21, 2021 to May 4, 2021).

### 3.2 Study population

The survey will use a convenient sampling and snowball strategies, and any health care workers (clinicians, nurses, and allied health staff [dentists, pharmacists, psychologists, occupational therapists, physiotherapists, case managers, and medical social workers]) working at the hospital and the general population in Thailand will be eligible for the online survey. The inclusion and exclusion criteria required for eligible participants are provided in Table 1.

**Table 1:**
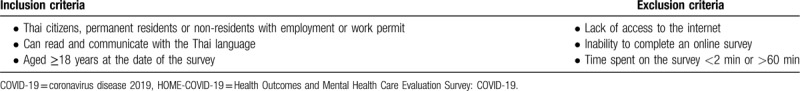
Eligibility criteria of the HOME-COVID-19 survey.

### 3.3 Online survey conduct

With respect to the physical distancing strategy and minimize face-to-face interaction, we developed an online questionnaire via the SurveyMonkey (https://www.surveymonkey.com) that limits 1-time participation per unique internet protocol (IP) address. The researcher team will provide the relevant link or the Quick Response code (QR code) to all potential participants. For healthcare workers, all staff working at the hospital will be invited to participate in the online survey via the personal contact and healthcare community social media networks (email, LINE, Group of Messenger, Healthcare Professional Association) in Thailand. A convenience and snowball sampling strategy will be applied to recruit the general population through various social media networks including public websites, Facebook, LINE, Twitter, and Instagram. No reimbursement, other gifts, or payments will be offered for completing the questionnaire.

### 3.4 Study instrument and questionnaire design process

The general population will complete a set of questionnaires, about participant characteristics and specific tool regarding the mental health and psychosocial question. Also, health care workers will complete a separate set of questions. The survey questionnaire, written in Thai, comprised 13 main components. There are different types of questions in the questionnaire including (yes/no) questions, the Likert scale, multiple-choice questions, and open-ended questions.

The questionnaire will be carefully revised by a panel of health care professionals that include 3 epidemiologists, 2 psychiatrists, 1 social scientist, and 2 hospital directors. The questionnaire will be further validated by a pilot survey of 30 health care professionals and 30 general populations. This validation process is used to measure the time needed to complete the questionnaire and assure that all the questions and sections of the questionnaire are phrased clearly and appropriately for comprehension.

Baseline sociodemographic characteristics include age, sex, educational level, marital status, religion, occupation/profession status, the region of residence/hospital, living status, number of a household family member, monthly income, job/income loss related to COVID-19 outbreak, financial problems, reimbursement schemes, comorbidities, media exposure, working from home information, quarantine/isolation information, willingness to quarantine/work during COVID-19 outbreak, global rating scale on the degree of fear to COVID-19, self-rated level of preparedness, and resilience capability against the COVID-19 outbreak. For health care workers, a constructed questionnaire is carried out using the established framework of hospital disaster resilience through existing literature^[4,5]^ and consultation with experts. These included the availability and adequacy of personal protective equipment and detailed information on hospital-level or capability related to COVID-19 outbreak and management.

A set of measurement tools for mental health and psychosocial problems for each subcohort are described in Table 2. We will focus on symptoms of depression,^[6]^ anxiety,^[7]^ stress,^[8]^ resilient copings,^[9]^ neuroticism,^[10]^ perceived social support,^[11]^ well-being,^[12]^ somatic symptoms,^[13]^ insomnia,^[14]^ burnout (for health care workers),^[15]^ and public stigma toward COVID-19 infection (for the general population), using a Thai version of validated measurement tools or our team development version tools. Detail of participants’ timeline and assessment for each wave of the survey is illustrated in Table 3.

**Table 2:**
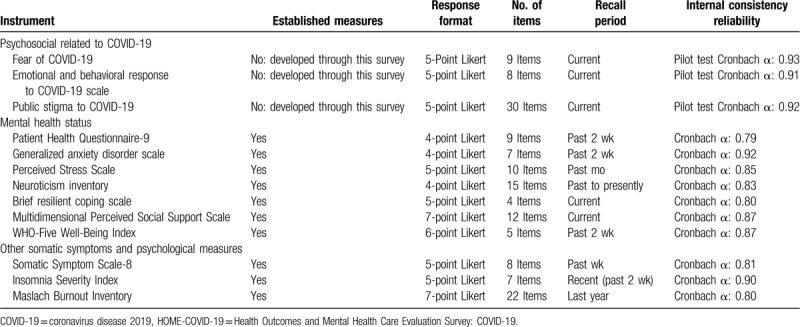
Measurement tools of the HOME-COVID-19 survey.

**Table 3:**
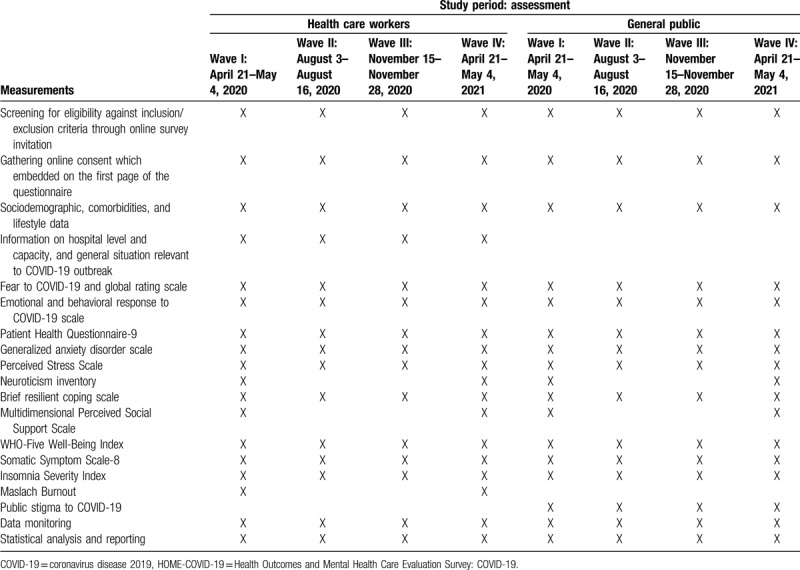
Schedule of measurement for each wave of the HOME-COVID-19 survey.

## 4 Statistical analysis plan

### 4.1 Sample size and power calculation

The target sample size of participants was estimated based on the previous studies among health care workers or general population under the COVID-19 outbreak: prevalence of mental health problems (depression, anxiety, stress, and psychological distress) with a range of 5.2% to 71.5%^[16–25]^ and 3.3% to 75.5%^[20,22,23,26–31]^ for health care workers and the general population, respectively. To compensate for a design effect of 2.0 and a completed responses rate of 60%, a target of at least 1182 health care workers and 1310 general populations is required to complete the online survey for each wave of the survey to ensure a power of 80% and a 0.05 type I error. However, there will be no restriction on the number of participants in this online survey.

### 4.2 Data management

The collected data will be organized by the Google Form and collected in an Excel spreadsheet. The online survey will be completely anonymous and uploaded to a Google Drive encrypted by a password. Only the management team can access all data. Data entered into the Google Form will be quality-checked by a researcher to ensure accuracy. All steps involved in the approach to data management will be independently conducted by 2 data administrators from the Pharmacoepidemiology and Statistics Research Center (PESRC).

### 4.3 Statistical analysis

Data synthesis will be conducted separately for health care workers and the general population. Descriptive data statistic will be summarized as number (percentage) or mean ± standard deviation, or median with an interquartile range as suitable. Differences in responses with respect to baseline characteristics will be assessed using Fisher exact test and unpaired *t* test or Wilcoxon rank-sum test for categorical and continuous data, respectively.

For all estimates of prevalence, we will weight data for all wave surveys (wave I, II, III, and IV) analyses under the complex design of the survey—weighting adjusted for noncompleted responses survey and national rates of unemployment at the time of each survey. Population control totals for age by sex and region, and the rate of internet use will be obtained from the National Statistic Office, Ministry of Information and Communication Technology, Thailand. The estimates of the prevalence of mental health status will be calculated and reported along with the 95% confidence intervals (CIs). Subgroup analyses stratified by key characteristics (eg, age, sex, region, working position [frontline or nonfrontline], hospital type, and region of residence) will also be done to analyze the proportion differences.

Moreover, an online wave II, III, and a 1-year follow-up survey will be compared to assess the long-term responses and resilience capability on the prevalence of mental health status among health care workers and the general population. Nonoverlapping 95% CIs are indicating statistical differences in the prevalence between the survey wave or subgroups, with the *P* values that are based on the trend tests. The absolute change in prevalence will be calculated with the formula: Δ = (1/duration between survey) × (p_last_ – p_initial_), with variance estimated as (var [p_last_] + var [p_initial_])/(duration between survey), where p_last_ and p_initial_, stand for the last prevalence and initial prevalence, respectively. Trend analysis will be performed to identify the effect of overtime across the study time frame.

For the survey—wave I, the crude association between participant characteristics will be assessed through the univariable logistic regression models to identify the candidate risk factors of early mental health problems. Subsequently, risk factors with a *P* value <.100 were then included in the multivariate logistic regression analysis with the stepwise backward method. The final model was also determined for multicollinearity by investigation of the variance inflation factors of the risk factors within the multivariable model. The effect estimates of the final risk factors model for mental health problems will be expressed as odds ratios (ORs) with corresponding 95% CIs.

For the repeated cross-sectional survey, we will combine the data from the wave I to wave IV survey to analyze changes in the mental health status. We will perform multilevel logistic regression models with random intercepts to explore associations with individual-level and region-level/hospital-level predictors. The survey wave will be analyzed as a level 1 variable with a fixed effect to distinguish between study survey waves across the study time frame.

To improve the generalizability of study findings, we also plan to perform an ancillary systematic review and meta-analysis by incorporating data from our findings to all available evidence regarding the mental health and psychosocial problems among health care workers and the general population. The prespecified protocol for systematic review has been registered in the International Prospective Register of Systematic Reviews (PROSPERO: registration number, CRD42020177120). Globally, the mental health problems attributable to the general population during the COVID-19 pandemic will be estimated by multiply the pooled prevalence of our findings against the United Nations’, world population report.

Missing data or noncompleted responses will be excluded from the primary analyses. However, a multiple imputation method will be performed in the sensitivity analysis. All analyses will be performed using Stata software version 14.0 (StataCorp, LP) and Microsoft Excel version 2016. They will be 2-tailed and *P* value <0.05 will be considered statistically significant.

## 5 Ethics and dissemination

### 5.1 Ethics approval and consent to participate

Ethics approval has been granted for the study by the Committee of Research Ethics in Faculty of Public Health (ET010/2020) and Faculty of Pharmacy (23/2563), Chiang Mai University. This study protocol will be performed according to the Declaration of Helsinki. All protocol amendments will be submitted to the ethics committees for approval. The protocol version 2–2020, dated April 23, 2020 was used to prepare this manuscript.

All respondents are required to fill a written informed consent (embedded on the first page of the survey questionnaire) before participating in this survey. If the participant answers “Yes” to the first question of the form, they automatically agree to participate and will be the survey. By using the skip-logic survey method, users who disagree with the informed consent question will be conducted to the end of the survey. No respondent is forced to participate in the survey and their participant is based on their agreement that can be withdrawn at any time. All participants have the right to leave a specific question unanswered or withdraw from the survey any time if they feel uncomfortable answering any question. Moreover, no one even the research team will know individual answers to this questionnaire.

### 5.2 Autonomy and confidentiality

All participants have the right to leave a specific question unanswered or withdraw from the survey any time if they feel uncomfortable answering any question. Moreover, no one even the research team will know individual answers to this questionnaire. All data are anonymous and are not able to identify the participants. The collected electronic data will remain confidential and only authorized team members will have access to it. Furthermore, data will be completely encrypted and coded for use mainly in statistical analysis using computer software.

### 5.3 Dissemination of results

Our findings will be disseminated through public, scientific, and professional meetings, and publications in peer-reviewed journals. Summary results in the lay written manner will also be available to the public. The investigators commit to reporting the results in line with the Strengthening the Reporting of Observational Studies in Epidemiology (STROBE) Statement: guidelines for reporting observational studies^[32]^ and Improving the quality of Web surveys: the Checklist for Reporting Results of Internet E-Surveys (CHERRIES).^[33]^

## 6 Discussion

Since the end of December 2019, the Chinese city of Wuhan has reported novel pneumonia caused by coronavirus disease 2019 (COVID-19) pneumonia outbreak (previously referred to as “Wuhan pneumonia” or 2019 Novel Coronavirus [2019-nCoV]).^[34]^ Due to potentially serious health outcomes, from January 23, 2020, Wuhan and other regions in mainland China have adopted strict quarantine measures to control the widespread of the COVID-19 outbreak.^[34]^ Subsequently, person-to-person transmission has been recorded outside mainland China, which is spreading domestically and internationally. On January 30, 2020, the World Health Organization (WHO) declared the global COVID-19 outbreak a public health emergency concern, consequently characterizes as a pandemic on March 11, 2020. As of April 26, 2020—situation report-97—a total of 2,810,325 confirmed cases with 193,825 confirmed deaths have been documented globally.^[35]^

The negative emotion and psychosocial distress may occur among frontline health care workers who have directly involved with the COVID-19 patients, which characterized by unintentional missing, trigger events considered into the mistake, and delay treatment owing to the communication failure. A cross-sectional study of 1257 health care workers who treated patients exposed to COVID-19 in multiple regions of china (including Wuhan) by Lai et al, 2020,^[16]^ revealed that participants reported symptoms of depression 50.4%, anxiety 44.6%, insomnia 34.0%, and distress 71.5%. Not surprisingly, frontline health care workers were associated with a higher risk of depression (OR, 1.52; 95% CI, 1.11–2.09; *P* = .010), anxiety (OR, 1.57; 95% CI, 1.22–2.02; *P* < .001), insomnia (OR, 2.97; 95% CI, 1.92–4.60; *P* < .001), and distress (OR, 1.60; 95% CI, 1.25–2.04; *P* < .001).

The affected people were also subject to societal rejection, discrimination, and stigmatization. Evidence suggests that the psychological impact of quarantine is wide-ranging, substantial, and can be long-lasting, including post-traumatic stress symptoms, confusion, and anger.^[36]^ A wide range of psychological and nonpsychological outcomes was reported and has negative consequences for segregated patients among isolation hospitalized patients who are infectious to contain the risk of infection.^[37]^ Moreover, loneliness, anger, frustration, and boredom could be experienced by individuals in the quarantine period. Interestingly, the previous report among the exposed individuals in the quarantine period with the Middle East respiratory syndrome coronavirus (MERS-CoV) outbreak in Korea found that 7.6% and 16.6% expressed anxiety and feelings of anger symptoms, respectively.^[38]^

To our knowledge, this is the first prospective, a repeated cross-sectional study among health care workers and the general population in Thailand. Given the limited evidence at a national level, our study will comprise an early- and long-term mental health status and responses to the COVID-19 outbreak in Thai health care and the general population. Our findings will also plan to perform an ancillary analysis by incorporating all available evidence to further systematic review and meta-analysis. However, as this study leverages online survey data, thereby, responses rates and incomplete information in some questionnaires may limit our findings.

## 7 Conclusion

This prospective repeated cross-sectional study, the national-based online survey will report the prevalence of mental health and psychosocial problems among health care workers and the general population in Thailand during the time of the COVID-19 pandemic. Findings from our study can aid health care professionals, public health officials, and public society by quantifying and identify factors that may accelerate or mitigate the negative impact of the COVID-19. This information can inform the design of strategies for public policy and cope with mental health and psychosocial life events. Our findings will disseminate in the forms of public and scientific meetings along with the peer-reviewed publication.

## Author contributions

**Conceptualization:** Surapon Nochaiwong, Chidchanok Ruengorn, Nahathai Wongpakaran, Tinakon Wongpakaran

**Data curation:** Ratanaporn Awiphan, Yongyuth Ruanta, Waraporn Boonchieng, Sirisak Nanta, Woravut Kowatcharakul, Wanida Pumpaisalchai, Penkarn Kanjanarat, Pajaree Mongkhon

**Formal analysis:** Surapon Nochaiwong, Chidchanok Ruengorn, Nahathai Wongpakaran, Tinakon Wongpakaran

**Funding acquisition:** Surapon Nochaiwong

**Investigation:** Surapon Nochaiwong, Chidchanok Ruengorn, Nahathai Wongpakaran, Tinakon Wongpakaran

**Methodology:** Surapon Nochaiwong, Chidchanok Ruengorn, Kednapa Thavorn, Brian Hutton

**Supervision:** Surapon Nochaiwong

**Writing – original draft:** Surapon Nochaiwong,

**Writing – review & editing:** all authors
